# Body image and mental health in women with polycystic ovary syndrome–a cross-sectional study

**DOI:** 10.1007/s00404-024-07913-4

**Published:** 2025-02-13

**Authors:** Konstantin Hofmann, Claire Decrinis, Norman Bitterlich, Katharina Tropschuh, Petra Stute, Annette Bachmann

**Affiliations:** 1 Department of Obstetrics and Gynecology, University Medical Center of Johannes Gutenberg University Mainz, Mainz, Germany; 2 King’s Fertility, Fetal Medicine Research Institute, London, UK; 3 Department of Obstetrics and Gynecology, University of Freiburg Medical Center, Freiburg, Germany; 4 Freelance Statistician “Analyzing, Consulting, Training”, Chemnitz, Germany; 5 Department of Obstetrics and Gynecology, Klinikum Rechts Der Isar, School of Medicine and Health, Technische Universität München, Munich, Germany; 6 Department of Obstetrics and Gynecology, University Clinic Inselspital Bern, Bern, Switzerland; 7 Department of Obstetrics and Gynecology, Division of Gynecological Endocrinology and Reproductive Medicine, University Medicine, Johann Wolfgang Goethe University, Frankfurt, Germany

**Keywords:** PCOS, Body image, Health-related quality of life, Depression, Anxiety, Obesity

## Abstract

**Purpose:**

Polycystic Ovary Syndrome (PCOS) is a multifaceted endocrine-metabolic condition affecting around 5–15% of women globally. Despite its prevalence and diverse impact, the psychological aspect of PCOS is often underestimated in clinical settings, leading to significant distress among affected individuals. This study aimed to explore the extent of body image perception disorders, psychological comorbidities, and their influence on the health-related quality of life (HRQOL) in women with PCOS. Additionally, we focused on measuring the impact of factors associated with PCOS, particularly obesity, to gain a more comprehensive understanding of their effects.

**Methods:**

An online survey was distributed anonymously to gynecologists, hospitals, and women’s clinics across Austria, Germany, and Switzerland, as well as through social media platforms to connect with women with PCOS. The survey was conducted from November 14, 2023, to February 05, 2024. HRQOL, anxiety/depression levels, body image and self-esteem were assessed employing the Modified-PCOS-Questionnaire (MPCOSQ), Hospital Anxiety and Depression Scale (HADS), Multidimensional Body-Self Relations Questionnaire Appearance Scales (MBSRQ-AS) and Rosenberg Self-Esteem Scale (RSE) respectively. Identification of potential confounding variables relied on their plausibility and association with the estimate. Adjusted odds ratios and their respective 95% confidence intervals were computed through regression analysis.

**Results:**

587 participants fully completed the questionnaire. The study participants were on average 32.5 ± 5.9 years old and had a BMI of 31.3 ± 7.8 kg/m^2^. In this study, 84.5% of all PCOS patients exhibited pathological scores in the MBSRQ-AS Appearance Evaluation, 83.8% in Body Areas Satisfaction, and 67.5% in Overweight Preoccupation. Half of the participants (46.7%) showed significantly poor results in the RSE, indicating low self-esteem. A notable portion of the study participants displayed elevated HADS scores, which supports a higher rate of distress in PCOS patients. (HADS-Anxiety > 8: 75.0% (440); HADS-Depression > 8: 57.6% (338). Examining the HRQOL of PCOS patients revealed that all average scores of the subscales, except for the Acne subscale and MPCOSQ-Total (3.6 ± 1:0), were situated in the lower half, indicating diminished HRQOL. The linear regression revealed that pathological values in the MBSRQ-AS, indicating impaired body image, were associated with low scores in the MPCOSQ subscales and HADS scales, suggesting impaired HRQOL and a higher rate of distress in PCOS patients. Furthermore, the multivariate analysis showed a statistically significant link between adverse body perception and HRQOL, as well as distress among PCOS patients in this study group. [multivariate HADS-Anxiety: MBSRQ-AS Body Areas Satisfaction (*B*: − 2.10; CI: − 3.88; − 0.33; *p* = 0.02) HADS-Depression MBSRQ-AS Body Areas Satisfaction (*B*: − 1.92; CI: − 3.59; − 0.26; *p* = 0.02)].

**Conclusion:**

Our findings reveal that obesity and a negative body image are interconnected factors that adversely affect both HRQOL and mental health in women with PCOS. Healthcare professionals should recognize the negative effects of obesity and poor body image in patients with PCOS and proactively provide effective treatment options.

## What does this study add to the clinical work

**Table:** 

This study highlights the interconnected impact of obesity and negative body image on the mental health and quality of life of women with PCOS, emphasizing the high prevalence of psychological distress in this population. Clinicians should consider body image concerns and obesity as key factors in the management of PCOS to improve overall patient well-being.	

## Introduction

Polycystic Ovary Syndrome (PCOS) is a multifaceted endocrine-metabolic condition that impacts around 5–15% of women during reproductive age globally [1, 2]. Therefore, it stands as the prevailing endocrine disorder among premenopausal women on a global scale [3].

Women with PCOS exhibit various manifestations of the disease, which significantly complicates uniform management. Five major areas of influence can be identified, which may be affected to varying degrees. These include body image (acne, hirsutism, alopecia, overweight), endometrium (oligo-/amenorrhea, endometrial carcinoma), metabolism (insulin resistance, metabolic syndrome, type 2 diabetes mellitus, cardiovascular risk factors), reproductive health (infertility, pregnancy complications), and mental health (body image, anxiety, depression, eating disorders, sleep disorders) [4, 5]. The psychological aspect is often overlooked in clinical practice, despite patients often experiencing significant distress [6].

Obesity is a common comorbidity in women with PCOS and significantly influences their physical and mental health outcomes [4]. Obesity is strongly linked to insulin resistance, resulting in hyperinsulinemia, a common characteristic in women with PCOS [7]. In PCOS patients, excess weight exacerbates hormonal imbalances, contributing to symptoms like irregular menstruation, hyperandrogenism, and insulin resistance, all of which compound the challenges of managing the syndrome [7]. Additionally, obesity is closely linked to negative body image perceptions in this population, leading to decreased body dissatisfaction. A meta-analysis by Davitadze et al., which included studies using the MBSRQ-AS as a measurement tool and involved 918 women with PCOS and 865 women without PCOS, found that women with PCOS have significantly greater body image concerns compared to women without PCOS [8].

These factors collectively impact quality of life, as body image concerns are often coupled with psychological comorbidities, including depression and anxiety, which are more prevalent in women with PCOS than in the general population. In an Australian longitudinal study, the prevalence of depression was up to 27.3% in PCOS patients compared to 18.8% in the control group, and 50% for anxiety including anxiety disorders compared to 29.2% in the control group [9]. A meta-analysis of 30 cross-sectional studies involving patients from 10 countries yielded a similar result: the risk of depressive symptoms is increased by 3.78-fold, while the risk of anxiety and anxiety disorders is increased by 5.62-fold compared to the control group [10].

In this study, it was examined whether the prevalence of body image perception disorders, psychological comorbidities (depression, anxiety) in women with PCOS was higher than currently assumed, and as a result, the HRQOL of those affected was compromised.

Given the broad and significant impact of PCOS on various aspects of health, the interplay between body image concerns and psychological health in PCOS patients demands further exploration. Especially the high prevalence of obesity among women with PCOS exacerbates the already challenging symptoms of the condition, particularly by worsening insulin resistance and hyperandrogenism, which in turn intensify body image issues. Our study seeks to further investigate this connection to understand better how body image disturbances may contribute to or exacerbate psychological symptoms, ultimately affecting the overall HRQOL for women with PCOS.

## Methods

This study was a non-interventional cross-sectional study. Data collection was conducted through an online questionnaire using the SoSciSurvey software from November 14 2023 to February 05, 2024 [11].

For the recruitment of the study population, flyers with the link to the survey were distributed to practicing gynecologists, hospitals, and womens clinics, with the request to pass them on to affected individuals or to display them in waiting rooms. Additionally, patients who may be missed through the methods were to be reached through social media or internet forums. Recruitment took place in Switzerland, Austria, and Germany.

### Inclusion and exclusion criteria

All women with PCOS were eligible to participate. The PCOS diagnosis was based on the criteria of the European Society of Human Reproduction and Embryology (ESHRE) and the PCOS phenotype were determined from the self-reported information.

Women who did not meet the diagnostic criteria for PCOS according to the ESHRE recommendation, as well as those whose thelarche occurred less than three years ago, pregnant, or lactating women, and those who reported never having had menstruation, were not included in the study. Additionally, a differential diagnosis not compatible with PCOS, such as congenital adrenal hyperplasia (CAH) or prolactinoma, resulted in exclusion. Since the survey was conducted as a German-language online questionnaire, sufficient knowledge of German and internet access were required.

Participants had to be over 18 years old, and their participation in the study had to be voluntary. The inclusion and exclusion criteria were checked at the beginning of the online questionnaire. If a participant met an exclusion criterion, the survey was terminated, and the participant was informed that they did not belong to the sought-after population**.**

### Psychosocial and clinical parameters

#### Body image

The Multidimensional Body-Self Relations Questionnaire (MBSRQ) assesses body image as a multidimensional construct within 34 items. The Appearance Scales (AS) of the MBSRQ (Appearance Evaluation, Appearance Orientation, Body Areas Satisfaction, Overweight Preoccupation, Self-Classified Weight) are subscales for assessing the presence of body image perception disorders and their severity. (MBSRQ-AS).

The mean norms for the MBSRQ-AS for women are [8, 12]:

- Appearance Evaluation: a seven-item scale of personal satisfaction with one’s physical appearance, elevated scores signify positive and positive perceptions of physical appearance and attractiveness (cut-off: 3.36).
- Appearance Orientation: a 12-item scale of personal investment in grooming and appearance presentation, elevated scores indicate elevated levels of focus on appearance and extensive grooming (cut-off 3.91).
- Overweight Preoccupation: a four-item scale of fat anxiety, weight vigilance, dieting, and eating restraint, elevated scores indicate elevated fat anxiety and levels of dieting behavior (cut-off 3.03).
- Body Areas Satisfaction: a nine-item scale that evaluates satisfaction with specific body areas and attributes (e.g., face, weight, and muscle tone), elevated scores indicate satisfaction with subjective body appearance (cut-off 3.23).
- Self-Classified Weight: a two-item scale that indicates the individual’s perception of their weight ranging from “I think I am” to the label of their weight as perceived by others. Scores vary from one—indicating a perception of being very underweight—to five—suggesting a perception of being very overweight. Elevated scores reflect a strong subjective and internalized perception of oneself as obese, while lower scores indicate a perception of oneself as thin. (cut-off 3.57).

In this study, the German version of the MBSRQ-AS was used [13].

#### Health-related quality of life

The PCOSQ was employed for evaluating HRQOL. It comprises 26 items addressing various symptom domains relevant to PCOS, such as emotions, body hair, weight, infertility, and menstruation. Responses were rated on a Likert scale from one to seven, with seven representing the most significant impairment [14].

Recently, four additional questions related to the “acne” subscale have been incorporated into the Modified PCOS-Questionnaire (MPCOSQ), which has undergone validation in various languages [15–17].

#### Mental state

Anxiety and depression were assessed using the Hospital Anxiety and Depression Scale (HADS). This self-report instrument comprises two subscales, one for anxiety and the other for depression, each consisting of seven questions. Each item offers four responses, ranging from zero (no symptoms) to three (maximum symptoms). Elevated scores indicate heightened levels of anxiety or depression. The cutoff point for both symptom domains is a score of eight or higher. Scores between eight and ten indicate mild anxiety or depression, 11–14 indicate moderate levels, and 15–21 indicate severe anxiety or depression [18].

The “Rosenberg Self-Esteem Scale” (RSE) was used to evaluate global self-esteem. It consists of ten statements related to self-worth, self-acceptance, and overall feelings of adequacy. Respondents indicate their level of agreement or disagreement with each statement, typically using a four-point Likert scale ranging from “strongly agree” to “strongly disagree.” The scale spans from 10 to 40, where scores falling between 20 and 30 are considered typical, while scores below 20 indicate lower levels of self-esteem [19, 20].

#### Hyperandrogenism

To assess hirsutism, patients answered the Ferriman-Gallwey questionnaire. A score above seven was considered pathological hirsutism [21]. The Ludwig classification was utilized to evaluate alopecia as a consequence of PCOS-triggered androgenization [22]. The participants were queried about their current experience with acne.

### Statistical analysis

The results were compared to values reported in existing literature. A sample size of 200 participants was deemed sufficient to statistically detect differences ranging from at least 7% (when occurrence rates in the general population are below 5%) to at least 11% (when occurrence rates in the general population are around 20%) with 80% power for binomial proportions. For changes in point scores (ordinal or cardinal scaled), a sample size of 200 participants could detect small effects with Cohen’s effect sizes of at least 0.25 with 80% power. To address the influence of multiple testing on four co-primary endpoints the Holm-Bonferroni correction was used.

The data underwent exploratory data analysis and descriptive statistical analysis such as mean ± standard deviation, frequency, and percentages using “IBM SPSS Statistics Version 29”.

Linear regression analyses were employed to assess the association of body image on both the total score and subscales of MPCOSQ, as well as on mental state. Variables of the subscales of the MBSRQ-AS and with *p*-values below 0.05 in the linear regression model were subsequently included in the final multivariate regression model using backward stepwise selection to determine their independence.

Before inclusion in the multivariate model, parameters underwent correlation checks. If the Spearman correlation coefficient exceeded 0.7, parameters were not simultaneously included in the model to avoid multicollinearity. Confounding variables were chosen from a set of clinically plausible factors, including age, BMI, diabetes mellitus, insulin resistance, hormone medication, menopausal status, desire for children, fertility problems, currently undergoing fertility treatment, PCOS-type, highest level of education, employment status, relationship status, Ludwig Score and Ferriman-Gallwey-Score. Model adjustment was performed using a change-in-estimate approach, where potential confounders were included in the final multivariate regression model if they altered the odds ratio (OR) by more than 10%. Effects were estimated using OR, *p*-value, and 95% confidence interval (CI).

### Ethical considerations

The research protocol was exempted from ethical review by the committee of Rhineland Palatinate and the Swiss Association of Research Ethics Committees, in accordance with German and Swiss legal statutes and regulations, which stipulate that studies involving anonymous data collection do not require formal ethical approval [2023–17312 (22nd December 2023)/BASEC Req-2023-01259 (1st November 2023)]. Both the study center and SoSci Survey adhered to German and European legislation regarding data protection. The study was conducted according to the requirements of the “Declaration of Helsinki” [23].

## Results

The questionnaire received 1786 responses, and although 778 individuals participated in the survey, the questionnaire was completed in full 690 times. 60 participants were excluded because of pregnancy or lactation, eight because less than three years have passed since thelarche, four participants stated that they have not yet had menstruation, and in 32 cases, they did not meet the ESHRE criteria for PCOS. The following data shows the results of the 587 fully completed questionnaires.

### Characteristics of the cohort

The average age of the study participants was 32.5 ± 5.9 years. The BMI was elevated (31.25 ± 7.8 kg/m^2^) and the prevalence of insulin resistance was 45.1%. The Ferriman-Gallwey Score showed increased hirsutism with an average of 14.1 ± 6.6 and a Ludwig score for alopecia of 1.0 ± 0.2 (Table 1).

**Table 1:** Characteristics of the study population

	Overall
Age (years)	Mean ± SD: 32.5 ± 5.9
Weight (kg)	88.7 ± 23.5
BMI (kg/m^2^)	31.3 ± 7.8
Diabetes mellitus	No 2.7% (16)
	Yes 97.3 (571)
Insulin resistance	Yes 45.1% (265)
	No 54.9% (322)
Currently under metabolic medication	Yes 41.7% (245)
	No 58.3% (342)
Currently under hormone medication	Yes 15.7% (92)
	No 84.3% (495)
Menopause status	Premenopausal: 96.9% (569)
	Postmenopausal: 3.1% (18)
Current desire to have children	Yes 43.8% (257)
	No 56.2% (330)
Fertility-problems (Have you ever tried to get pregnant for more than 1 year?)	Yes 48.6% (285)
	No 51.4% (302)
Currently undergoing fertility treatment	Yes 19.4% (114)
	No 80.6% (473)
PCOS-type	A 75.5% (443)
	B 7.8% (46)
	C 14.7% (86)
	D 1.9% (11)
Highest level of education	Currently attending school: 0.2% (1)
	School-leaving qualification: 6.3% (37)
	Secondary school certificate: 25.2% (148)
	Polytechnic secondary school: 0.7% (4)
	Advanced college certificate: 14.5% (85)
	Abitur: 17.9% (105)
	University: 31.7% (186)
	Other: 3.6% (21)
Employment	Yes 87.7% (515)
	No 12.3 (72)
Partnership	Yes 84.7% (497)
	No 15.3% (90)
Ludwig-score	Mean ± SD: 1.0 ± 0.2
Ferriman-Gallwey-score	Mean ± SD: 14.1 ± 6.6

*BMI* Body Mass Index, *PCOS* Polycystic Ovary Syndrome, *SD* standard deviation), absolute number of study participants is indicated in brackets next to the percentages; The World Health Organization defines Body Mass Index (BMI) ≤ 24.9 kg/m^2^, 25–29.9 kg/m^2^, ≥ 30 kg/m^2^ and ≥ 35 kg/m^2^ as healthy weight, overweight and obesity I and II; “Currently under metabolic medication” was defined as the current intake of Metformin, Insulin, incretin mimetics, or other antidiabetic medications; Definition of PCOS-type: *A *= hyperandrogenism + ovulatory dysfunction + PCO-morphology; *B *= hyperandrogenism + ovulatory dysfunction; *C *= hyperandrogenism + PCO-morphology; *D* = ovulatory dysfunction + PCO-morphology

### Body image and self-esteem

The values of the MBSRQ-AS categories Appearance Evaluation, Body Areas Satisfaction, and Overweight Preoccupation were in the pathological range in this group. For Appearance Orientation and Self-classified Weight, average values were within the normal range (Table 2). The values of the RSE were below the cut-off point, with 15.0 ± 6.8 (Table 3).

**Table 2:** Proportion of patients with distorted body image; absolute number of study participants is indicated in brackets next to the percentages

MBSRQ-AS	Appearance evaluation Mean ± SD	Appearance orientation	Body areas satisfaction	Overweight preoccupation	Self-classified weight
Overall	2.3 ± 0.9 Pathological (< 3.36): 84.5% (496)	3.5 ± 0.7 Pathological (> 3.91): 28.3% (166)	2.6 ± 0.7 Pathological (< 3.23): 83.8% (492)	3.5 ± 1.0 Pathological (> 3.03): 67.5% (396)	4.2 ± 0.8 Pathological (< 3.57): 29.1% (171)

*MBSRQ-AS* Multidimensional Body-Self Relations Questionnaire–Appearance Scales

**Table 3:** Proportion of patients with decreased self-esteem in the Rosenberg Self-Esteem Scale (RSE)

RSE	< 20	Overall Mean ± SD
Overall	46.7% (274)	15.0 ± 6.8

Absolute number of study participants is indicated in brackets next to the percentages

### Health-related quality of life

**Fig. 1:**
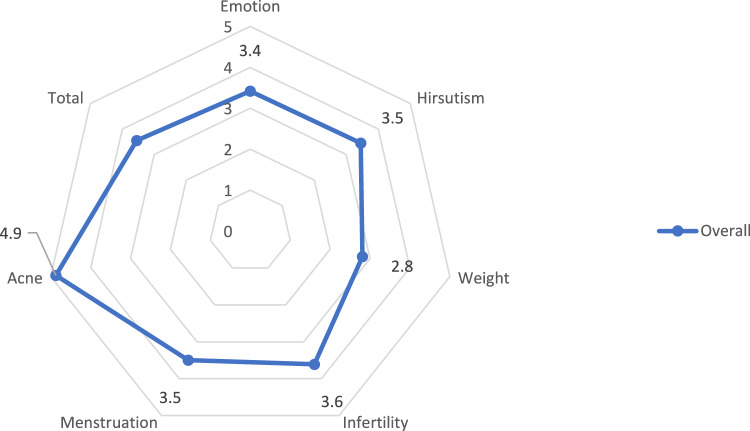
Health-related quality of life assessed with the Modified PCOS-Questionnaire (MPCOSQ)

The study population had low scores in all subscales and in the overall score. Only the acne subscale showed high values (Table 4; Fig. 1).

**Table 4:** Health-related quality of life assessed with the Modified PCOS-Questionnaire (MPCOSQ)

	Emotion Mean ± SD	Body hair	Weight	Infertility	Menstruation	Acne	Total
Overall	3.4 ± 1.1	3.5 ± 1.8	2.8 ± 1.8	3.6 ± 1.7	3.5 ± 1.2	4.9 ± 2.0	3.6 ± 1.0

#### Mental state

The study participants reported an average anxiety score of 10.3 ± 4.0. About 26.8% had mild, 38.7% moderate and 9.0% severe symptoms of anxiety (Tables 5).

**Table 5:** Proportion of patients with increased distress; absolute number of study participants is indicated in brackets next to the percentages

HADS	0–7 (normal)	8–10 (mild)	11–14 (moderate)	15–21 (severe)	Overall Mean ± SD
Anxiety-subscale	25.0% (147)	26.8% (157)	38.7% (227)	9.5% (56)	10.3 ± 4.0
Depression-subscale	42.4% (249)	29.6% (174)	22.7% (133)	5.3% (31)	8.3 ± 4.1

*HADS* Hospital Anxiety and Depression Scale

**Fig. 2:**
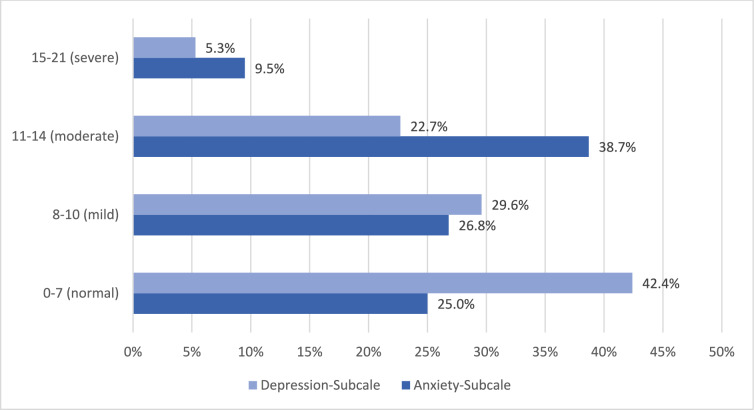
Results of the Hospital Anxiety and Depression Scale (HADS)

The mean depression score was 8.26 ± 4.1. In total. 29.6% reported mild, 22.7% moderate, and 5.3% severe symptoms of depression (Fig. 2).

### Association between body image, HRQOL and mental state

In the regression analysis, the subscales of the MBSRQ-AS exhibited a significant association with the subscales of the MPCOSQ and HADS. Potential confounders were also assessed for association with HRQOL and mental state and are listed in Table 6.

**Table 6:** Association between Health-related quality of life (HRQOL), anxiety, depression and body image and self-esteem; linear regression; p-values less than 0.05 are in bold

	MPCOSQ-Emotion	MPCOSQ-Body hair	MPCOSQ-Weight	MPCOSQ-Infertility	MPCOSQ-Menstruation	MPCOSQ-Acne	MPCOSQ-Total	HADS-Anxiety	HADS-Depression
Age	*B*: 0.25 (CI: 0.01; 0.04) ***p* < 0.001**	*B*: − 0.02 (CI: − 0.04; 0.01) *p* = 0.15	*B*: − 0.05 (CI: − 0.07; − 0.02) ***p* < 0.001**	*B*: 0.06 (CI: 0.04; 0.08) ***p* < 0.001**	*B*: 0.05 (CI: 0.03; 0.06) ***p* < 0.001**	*B*: 0.06 (CI: 0.03; 0.09) ***p* < 0.001**	*B*: 0.02 (CI: 0.00; 0.03) ***p* = 0.01**	*B*: − 0.01 (CI: − 0.06; 0.05) *p* = 0.78	*B*: 0.40 (CI: 0.04; 0.16) ***p* < 0.001**
BMI	*B*: − 0.02 (CI:− 0.04; − 0.01) ***p* < 0.001**	*B*: − 0.06 (CI: − 0.08; − 0.05) ***p* < 0.001**	*B*: − 0.14 (CI: − 0.16; − 0.13) ***p* < 0.001**	*B*: − 0.04 (CI: − 0.06; − 0.03) ***p* < 0.001**	*B*: − 0.00 (CI: − 0.02; 0.10) *p* = 0.67	*B*: 0.03 (CI: 0.01; 0.05) ***p* = 0.01**	*B*: − 0.04 (CI: − 0.05; − 0.03) *p*** < 0.001**	*B*: 0.07 (CI: 0.02; 0.11) ***p* = 0.002**	*B*: 0.11 (CI: 0.06; 0.15) ***p* < 0.001**
No diabetes mellitus	*B*: 0.30 (CI: − 0.23; 0.84) *p* = 0.265	*B*: 0.54 (CI: − 0.38; 1.45) *p* = 0.25	*B*: 0.75 (CI: − 0.15; 1.65) *p* = 0.10	*B*: 0.30 (CI: − 0.53; 1.14) *p* = 0.48	*B*: 0.18 (CI: − 0.41; 0.76) *p* = 0.56	*B*: − 0.47 (CI: − 1.44; 0.51) *p* = 0.35	*B*: 0.30 (CI: − 0.18; 0.77) *p* = 0.22	*B*: 0.05 (CI: − 1.94; 2.05) *p* = 0.96	*B*: 0.21 (CI: − 1.85; 2.26) *p* = 0.85
No insulin resistance	*B*: 0.24 (CI: 0.07; 0.42) ***p* = 0.01**	*B*: 0.50 (CI: 0.20; 0.79) ***p* = 0.001**	*B*: 0.95 (CI: 0.67; 1.24) ***p* < 0.001**	*B*: 0.32(CI: 0.05; 0.59) ***p* = 0.02**	*B*: 0.19 (CI: 0.00; 0.38) ***p* = 0.05**	*B*: − 0.03 (CI: − 0.35; 0.29) *p* = 0.86	*B*: 0.37 (CI: 0.22; 0.52) ***p* < 0.001**	*B*: − 0.64 (CI: − 1.29; 0.01) *p* = 0.06	*B*: − 0.98 (CI: − 1.65; − 0.31) ***p* = 0.004**
Currently not under hormone medication	*B*: − 0.08 (CI: 0.32; 0.16) *p* = 0.50	*B*: − 0.27 (CI: − 0.68; 0.14) *p* = 0.19	*B*: 0.36 (CI: − 0.05; 0.76) *p* = 0.08	*B*: − 0.71 (CI: − 1.08; − 0.34) ***p* < 0.001**	*B*: − 0.01 (CI: − 0.28; 0.25) *p* = 0.92	*B*: 0.04 (CI: − 0.40; 0.47) *p* = 0.88	*B*: − 0.10 (CI: − 0.31; 0.11) *p* = 0.36	*B*: − 0.22 (CI: − 1.12; 0.67) *p* = 0.62	*B*: 0.66 (CI: − 0.26; 1.58) *p* = 0.16
Premenopausal	*B*: 0.59 (CI: 0.13; 1.05) ***p* = 0.01**	*B*: 0.89 (CI: 0.11; 1.67) ***p* = 0.03**	*B*: 0.90 (CI: 0.13; 1.67) ***p* = 0.02**	*B*: 0.20 (CI: − 0.51; 0.92) *p* = 0.58	*B*: 0.04 (CI: − 0.47; 0.54) *p* = 0.89	*B*: 0.97 (CI: 0.13; 1.80) ***p* = 0.02**	*B*: 0.62 (CI: 0.21; 1.02) ***p* = 0.003**	*B*: − 0.49 (CI: − 2.20; 1.22) *p* = 0.57	*B*: − 1.10 (CI: − 2.86; 0.67) *p* = 0.22
No current desire to have children	*B*: 0.24 (CI: 0.06; 0.41) ***p* = 0.01**	*B*: − 0.01 (CI: − 0.31; 0.29) *p* = 0.96	*B*: − 0.20 (CI: − 0.49; 0.10) *p* = 0.19	*B*: 2.16 (CI: 1.95; 2.37) ***p* < 0.001**	*B*: 0.18 (CI: − 0.01; 0.37) *p* = 0.06	*B*: − 0.33 (CI: − 0.65: − 0.01) ***p* = 0.04**	*B*: 0.30 (CI: 0.14; 0.45) ***p* < 0.001**	*B*: 0.03 (CI: − 0.63;0.68) *p* = 0.93	*B*: − 0.24 (CI: − 0.91–0.44) *p* = 0.49
No fertility-problems	*B*: 0.08 (CI: − 0.10–0.25) *p* = 0.39	*B*: 0.23 (CI: − 0.06–0.53) *p* = 0.12	B: 0.43 (CI: 0.13–0.72) ***p* = 0.004**	B: 1.18 (CI: 0.93–1.44) ***p* < 0.001**	B: − 0.08 (CI: − 0.27–0.12) *p* = 0.43	B: − 0.34 (CI: − 0.66; − 0.02) ***p* = 0.04**	B: 0.23 (CI: 0.08; 0.39) ***p* = 0.003**	*B*: 0.16 (CI: − 0.49; 0.81) *p* = 0.63	*B*: − 0.60 (CI: − 1.26; 0.07) *p* = 0.08
Currently not undergoing fertility treatment	*B*: − 0.01 (CI: − 0.23; 0.21) *p* = 0.90	*B*: −0.49 (CI: − 0.86; 0.12) ***p* = 0.01**	*B*: − 0.40 (CI: − 0.77; − 0.03) ***p* = 0.03**	*B*: 1.66 (CI: 1.34; 1.97) ***p* < 0.001**	*B*: − 0.14 (CI: − 0.38; 0.10) *p* = 0.25	*B*: − 0.47 (CI: − 0.87; − 0.07) ***p* = 0.02**	*B*: − 0.01 (CI: − 0.21; 0.18) *p* = 0.90	*B*: 0.85 (0.03; 1.67) ***p* = 0.04**	*B*: 0.60 (CI: − 0.25; 1.44) *p* = 0.17
PCOS-type	*B*: 0.13 (CI: 0.02; 0.24) ***p* = 0.02**	*B*: 0.12 (CI: − 0.07; 0.30) *p* = 0.21	*B*: − 0.001 (CI: − 0.18; 0.18) *p* = 0.99	*B*: 0.20 (CI: 0.03; 0.36) ***p* = 0.02**	*B*: 0.13 (CI: 0.01; 0.25) ***p* = 0.03**	*B*: − 0.02 (CI: − 0.22; 0.17) *p* = 0.82	*B*: 0.09 (CI: 0.00; 0.19) ***p* = 0.049**	*B*: 0.05 (CI: − 0.35; 0.45) *p* = 0.81	*B*: 0.07 (CI: − 0.34; 0.48) *p* = 0.72
Highest level of education	*B*: 0.13 (CI: 0.08; 0.17) ***p* < 0.001**	*B*: 0.19 (CI: 0.11; 0.27) *p*** < 0.001**	*B*: 0.22 (CI: 0.14; 0.30) ***p* < 0.001**	*B*: 0.19 (CI: 0.11; 0.26) ***p* < 0.001**	*B*: 0.08 (CI: 0.02; 0.13) ***p* = 0.004**	*B*: − 0.06 (CI: − 0.14; 0.03) *p* = 0.19	*B*: 0.13 (CI: 0.09; 0.17) ***p* < 0.001**	*B*: − 0.19 (CI: − 0.37;; 0.01) ***p* = 0.04**	*B*: − 0.28 (CI: − 0.46; − 0.10) ***p* = 0.002**
Not employed	*B*: − 0.03 (CI: − 0.11; 0.05) *p* = 0.49	*B*: − 0.09 (CI: − 0.23; 0.05) *p* = 0.21	*B*: 0.06 (CI: − 0.08; 0.20) *p* = 0.39	*B*: 0.05 (CI: − 0.08; 0.18) *p* = 0.41	*B*: − 0.11 (CI: CI: − 0.20; 0.02) ***p* = 0.02**	*B*: 0.03 (CI: − 0.12; 0.19) *p* = 0.67	*B*: − 0.02 (CI: − 0.09; 0.06) *p* = 0.67	*B*: 0.17 (CI: − 0.14; 0.48) *p* = 0.29	*B*: 0.19 (CI: − 0.14; 0.51) *p* = 0.26
Not living in a partnership	*B*: 0.01 (CI: − 0.06; 0.07) *p* = 0.79	*B*: − 0.00 (CI: − 0.11; 0.11) *p* = 0.98	B: − 0.09 (CI: − 0.19; 0.02) *p* = 0.11	B: − 0.11 (CI: − 0.21; − 0.01) ***p* = 0.03**	*B*: 0.03 (CI: − 0.04; 0.10) *p* = 0.36	*B*: 0.12 (CI: 0.01; 0.24) ***p* = 0.04**	*B*: − 0.01 (CI: − 0.06; 0.05) *p* = 0.82	*B*: 0.11 (CI: − 0.13; 0.34) *p* = 0.38	*B*: 0.18 (CI: − 0.07; 0.42) *p* = 0.16
Ludwig-score	*B*: − 0.54(CI: − 1.05; − 0.02) ***p* = 0.04**	*B*: − 0.18(CI: − 1.03; 0.67) *p* = 0.68	*B*: − 0.75(CI: − 1.56; 0.07) *p* = 0.07	*B*: − 0.76(CI: − 1.65; 0.14) *p* = 0.10	*B*: 0.05(CI: − 0.55; 0.64) *p* = 0.88	*B*: − 0.04(CI: − 1.04; 0.97) *p* = 0.95	*B*: − 0.40(CI: − 0.86; 0.07) *p* = 0.10	*B*: 1.53(CI: − 0.47; 3.53) *p* = 0.13	*B*: 1.38(CI: − 0.65; 3.41) *p* = 0.18
Ferriman-gallwey-score < 7	*B*: 0.36 (CI: 0.12; 0.61) ***p* = 0.004**	*B*: 1.99 (CI: 1.67; 2.31) ***p* < 0.001**	*B*: 0.60 (CI: 0.19; 1.01) ***p* = 0.004**	*B*: 0.20 (CI: − 0.19; 0.60) *p* = 0.32	*B*: 0.23 (CI: − 0.04; 0.50) *p* = 0.10	*B*: − 0.13 (CI: − 0.59; 0.33) *p* = 0.58	*B*: 0.57 (CI: 0.36; 0.77) ***p* < 0.001**	*B*: − 0.88 (CI: − 1.81; 0.04) *p* = 0.06	*B*: − 1.65 (CI: − 2.59; − 0.71) ***p* < 0.001**
M*B*SRQ-AS appearance evaluation < 3.36	*B*: 0.95 (CI: 0.72; 1.18) *p*** < 0.001**	*B*: 1.32 (CI: 0.92; 1.71) ***p* < 0.001**	B: 2.90 (CI: 2.57; 3.23) ***p* < 0.001**	B: 0.61 (CI: 0.23; 0.98) ***p* < 0.001**	*B*: 0.32 (CI: 0.06; 0.59) ***p* = 0.02**	*B*: 0.37 (CI: − 0.07; 0.81) *p* = 0.10	*B*: 1.13 (CI: 0.94; 1.32) ***p* < 0.001**	*B*: − 2.67 (CI: − 3.54; − 1.80) ***p* < 0.001**	*B*: − 3.82 (CI: − 4.69; − 2.95) ***p* < 0.001**
M*B*SRQ-AS appearance orientation > 3.91	*B*: 0.44 (CI: 0.25; 0.63) ***p* < 0.001**	*B*: 0.10 (CI: − 0.23; 0.43) *p* = 0.55	*B*: 0.85 (CI: 0.54; 1.17) ***p* < 0.001**	*B*: 0.03 (CI: − 0.27; 0.33) *p* = 0.85	*B*: 0.20 (CI: − 0.01; 0.41) *p* = 0.06	*B*: 0.51 (CI: 0.16; 0.86) ***p* = 0.004**	*B*: 0.38 (CI: 0.21; 0.54) ***p* < 0.001**	*B*: − 0.99 (CI: − 1.71; − 0.28) ***p* = 0.01**	*B*: 0.16 (CI: − 0.58; 0.91) *p* = 0.67
MBSRQ-AS body areas satisfaction < 3.23	*B*: 1.06 (CI: 0.84; 1.28) ***p* < 0.001**	*B*: 1.34 (CI: 0.95; 1.72) ***p* < 0.001**	*B*: 2.66 (CI: 2.33; 3.0) ***p* < 0.001**	*B*: 0.56 (CI: 0.20; 0.93) ***p* = 0.003**	*B*: 0.43 (CI: 0.17; 0.69) ***p* = 0.001**	*B*: 0.43 (CI: 0.00; 0.86) ***p* = 0.049**	*B*: 1.14 (CI: 0.95; 1.33) ***p* < 0.001**	*B*: − 3.44 (CI: − 4.27; − 2.60) ***p* < 0.001**	*B*: − 3.94 (CI: − 4.79; − 3.09) ***p* < 0.001**
MBSRQ-AS overweight preoccupation > 3.03	*B*: 0.60 (CI: 0.42; 0.78) ***p* < 0.001**	*B*: 0,62 (CI: 0.31; 0.93) ***p* < 0.001**	*B*: 2.17 (CI: 1.91; 2.43) ***p* < 0.001**	*B*: 0.41 (CI: 0.12; 0.70) ***p* = 0.01**	*B*: 0.18 (CI: − 0.02; 0.39) *p* = 0.08	*B*: 0.12 (CI: − 0.22; 0.46) *p* = 0.48	*B*: 0.72 (CI: 0.57; 0.87) ***p* < 0.001**	*B*: − 1.72 (CI: − 2.40; − 1.04) ***p* < 0.001**	*B*: − 1.72 (CI: − 2.42; − 1.02) ***p* < 0.001**
M*B*SRQ-AS Self- classified weight < 3.57	*B*: 0.50 (CI: 0.32; 0.69) ***p* < 0.001**	*B*: 0.97 (CI: 0.65; 1.29) ***p* < 0.001**	*B*: 2.75 (CI: 2.52; 2.98) ***p* < 0.001**	*B*: 0.63 (CI: 0.33; 0.92) ***p* < 0.001**	*B*: 0.07 (CI: 0.14; 0.28) *p* = 0.52	*B*: − 0.19 (CI: − 0.54; 0.16) *p* = 0.29	*B*: 0.82 (CI: 0.67; 0.98) ***p* < 0.001**	*B*: − 1.09 (CI: − 1.80; − 0.38) ***p* = 0.003**	*B*: − 1.87 (CI: − 2.59; − 1.15) ***p* < 0.001**
RosenBerg self-esteem score > 20	*B*: 0.93 (CI: 0.77; 1.09) ***p* < 0.001**	*B*: 1.10 (CI: 0.82; 1.39) ***p* < 0.001**	*B*: 0.92 (CI: 0.64; 1.21) ***p* < 0.001**	*B*: 0.68 (CI: 0.42; 0.95) ***p* < 0.001**	*B*: 0.50 (CI: 0.31; 0.68) ***p* < 0.001**	*B*: 0.66 (CI: 0.35; 0.98) ***p* < 0.001**	*B*: 0.83 (CI: 0.69; 0.97) ***p* < 0.001**	*B*: − 3.52 (CI: − 4.41; − 2.94) ***p* < 0.001**	*B*: − 4.33 (CI: − 4.90; − 3.75) ***p* < 0.001**

(*B* regression coefficient, *BMI* Body Mass Index, *CI* confidence interval, *HADS* Hospital Anxiety and Depression Scale, *MBSRQ*− *AS* Multidimensional Body-Self Relations Questionnaire–Appearance Scales, *MPCOSQ* Modified PCOS-Questionnaire, *PCOS* Polycystic Ovary Syndrome)

A multivariate regression analysis was conducted for all subdomains of the MPCOSQ and HADS. Potential confounders were included in the model if they met the change in estimate criterion. Before inclusion, all parameters were checked for correlation, with none showing a correlation coefficient above 0.7. In all subscales of the MPCOSQ (except for MPCOSQ-Acne), a negative body image measured with the MBSRQ-AS showed an association with HRQOL. In the MPCOSQ-Total, MBSRQ-AS Body Areas Satisfaction (*B*: 0.49, CI: 0.10–0.87, *p* = 0.01), MBSRQ-AS Overweight Preoccupation (*B*: 0.27, CI: 0.04–0.49, *p* = 0.02), and MBSRQ-AS Self-Classified Weight (*B*: 0.66, CI: 0.43–0.89, *p* < 0.001) showed an association with HRQOL. In the HADS subscales HADS-Anxiety and HADS-Depression, only MBSRQ-AS Body Areas Satisfaction (HADS-Anxiety: *B*: − 2.10, CI: − 3.88; − 0.33, *p* = 0.02; HADS-Depression: *B*: − 1.92, CI: − 3.59; − 0.26, *p* = 0.02) was significant in the multivariate model (Table 7).

**Table 7:** Association between Health-related quality of life (HRQOL), anxiety, depression and body image and self-esteem; multivariate linear regression

	MPCOSQ-Emotion	MPCOSQ-Body hair	MPCOSQ-Weight	MPCOSQ-Infertility	MPCOSQ-Menstruation	MPCOSQ-Acne	MPCOSQ-Total	HADS-Anxiety	HADS-Depression
Variables included in the model	Age, no insulin resistance, premenopausal, PCOS-type, highest level of education, not employed, Ludwig-Score, FG < 7, RSE > 20	BMI, no insulin resistance, premenopausal, currently not undergoing fertility treatment, highest level of education, Ludwig-Score, FG < 7, RSE > 20	BMI, Ludwig-Score, FG < 7, RSE > 20	Age, BMI, currently not under hormone medication, no fertility problems, currently not undergoing fertility treatment, PCOS-type, highest level of education, partnership, Ludwig-Score, FG < 7, RSE > 20	Age, BMI, no insulin resistance, no fertility problems, currently not undergoing fertility treatment, PCOS-type, highest level of education, not employed, Ludwig-Score, FG < 7, RSE > 20	Age, BMI, no fertility problems, highest level of education, Ludwig-Score, RSE > 20	Ludwig-Score, FG < 7, RSE > 20	Age, BMI, currently not undergoing fertility treatment, not employed, Ludwig-Score, FG < 7, RSE > 20	Age, BMI, no insulin resistance, no fertility problems, highest level of education, not employed, Ludwig-Score, FG < 7, RSE > 20
Other stat. significant results of the multivariate model	Age: *B*: 0.02(CI: 0.00; 0.04) *p* = 0.03 Premenopausal: *B*: 0.77 (CI: 0.29; 1.26) *p* = 0.002 RSE > 20: *B*: 0.81 (CI: 0.58; 1.04) *p* < 0.001	FG < 7: *B*: 1.64 (CI: 1.13; 2.14) *p* < 0.001	BMI: *B*: − 0.04 (CI: − 0.04 (CI: − 00.06;− 0.02) *p* < 0.001 RSE > 20: *B*: 0.40 (CI: 0.13; 0.66) *p* = 0.004	Age: *B*: 0.09 (CI: 0.06; 0.16) *p* < 0.001 No fertility problems: *B*: 1.08 (CI: 0.66; 1.50) *p* < 0.001 Currently not undergoing fertility treatment: *B*: 1.40 (CI: 0.91; 1.90) *p* < 0.001 RSE > 20: *B*: 0.47 (CI: 0.10; 0.83) *p* = 0.01	Age: *B*: 0.05 (CI: 0.03; 0.08) *p* < 0.001 Not employed: *B*: − 0.14 (CI: − 0.27;− 0.01) *p* = 0.03 RSE > 20: *B*: 0.30 (CI: 0.00; 0.59) *p* = 0.047	RSE > 20: *B*: 0.92 (CI: 0.46; 1.38) *p* < 0.001	Age: *B*: 0.03 (CI: 0.01; 0.0) *p* = 0.002 FG < 7: *B*: 0.34 (CI: 0.06; 0.62) *p* = 0.002 RSE > 20: *B*: 0.54 (CI: 0.35; 0.73) *p* < 0.001	RSE > 20: *B*: − 3.02(CI: − 3.96;− 2.08) *p* < 0.001	Age: *B*: 0.09 (CI: 0.02; 0.16) *p* = 0.02 FG < 7: *B*: − 1.43 (CI: − 2.71;− 0.15) *p* = 0.03 RSE > 20: *B*: − 4.10 (CI: − 4.97;− 3.22) *p* < 0.001
Stat. significant results of the MBSRQ-AS	AE: *B*: 0.64 (CI: 0.17; 1.11) *p* = 0.01) AO: *B*: 0.32 (CI: 0.08; 0.56) *p* = 0.01 SCW: *B*: 0.37 (CI: 0.09; 0.65) *p* = 0.01	BAS: *B*: 0.77 (CI: 0.09; 1.45) *p* = 0.03 SCW: *B*: 0.52 (CI: 0.11; 0.93) *p* = 0.01	BAS: *B* 0.88 (CI: 0.33; 1.43) *p* = 0.002 OP: *B*: 1.02 (CI: 0.71; 1.33) *p* < 0.001 SCW: *B*: 1.65 (1.25; 2.04) *p* < 0.001	SCW: *B*: 0.57 (CI: 0.12; 1.02) *p* = 0.01	BAS: *B*: 0.71 (CI: 0.16; 1.27) *p* = 0.01	None	BAS: *B*: 0.49 (CI: 0.10; 0.87) *p* = 0.01 OP: *B*: 0.27 (CI: 0.04; 0.49) *p* = 0.02 SCW: *B* 0.66 (CI: 0.43; 0.89) *p* < 0.001	BAS: *B*: − 2.10 (CI: − 3.88;− 0.33) *p* = 0.02	BAS: *B*: − 1.92 (CI: − 3.59; − 0.26) *p* = 0.02
*R*^2^/adjusted *R*^2^/N	0.31/ 0.29/ 248	0.25/0.23/244	0.61/0.61/244	0.36/0.35/244	0.15/0.13/244	0.11/0.13/244	0.38/0.36/244	0.19/0.19/244	0.34/0.33/244

(*AE* MBSRQ-AS Appearance Evaluation, *AO* MBSRQ-AS Appearance Orientation, *BAS* MBSRQ-AS Body Areas Satisfaction, *B* regression coefficient, *BMI* Body Mass Index, *CI* confidence interval, *FG* Ferriman-Gallwey-Score, *HADS* Hospital Anxiety and Depression Scale, *MBSRQ-AS* Multidimensional Body-Self Relations Questionnaire–Appearance Scales, *MPCOSQ:* Modified PCOS-Questionnaire, *OP* MBSRQ-AS Overweight Preoccupation, *PCOS* Polycystic Ovary Syndrome, *RSE* Rosenberg Self-Esteem Scale, *SCW* MBSRQ-AS Self-classified Weight, *stat.* statistically)

## Discussion

Our findings indicated that a sizable portion of PCOS patients experienced a disrupted perception of their body image, reflected in both a negative assessment of their appearance and dissatisfaction with various body areas. Body image disturbances in PCOS patients often coexisted with reduced self-confidence, indicating that these women might experience negative emotions and diminished self-esteem. Additionally, a considerable number of participants exhibited elevated levels of distress. Specifically, 75% had elevated HADS-Anxiety scores, and 57.6% had elevated HADS-Depression scores.

Furthermore, our study demonstrated an association between body image, mental state, and HRQOL. Regression analysis indicated that pathological values in the MBSRQ-AS, reflecting impaired body image, were associated with low scores in the MPCOSQ subscales and high distress levels, suggesting impaired HRQOL among PCOS patients. Multivariate analysis confirmed the significant link between adverse body perception, HRQOL, and distress in this study group.

Consistent with our results, previous research has identified PCOS as an independent factor contributing to reduced HRQOL, often linked to negative body image [4, 24–26]. For example a case–control study by Alur-Gupta et al., which included 89 PCOS patients and 225 controls, found that individuals with PCOS reported lower satisfaction with body image. In line with our findings, these patients scored pathologically in MBSRQ-AS subcategories, with additional correlations observed between poor body image, lower quality of life, depression, and anxiety [27].

The accumulating evidence underscores the relationship between body image and mental well-being in PCOS, where negative body image impacts both physical and psychological dimensions of health. A negative body image impacts both physical and psychological well-being and has the potential to affect self-esteem, mood, sense of competence, as well as social and occupational functioning [4, 28]. The underlying cause is a complex system of pathological processes caused by PCOS.

PCOS patients may have a poor body image due to hormonal changes that lead to acne, alopecia, and excessive hair growth. These physical symptoms, along with the challenges of infertility and psychological burdens such as depression and anxiety, often impair self-confidence [29]. Additionally, factors independent of PCOS, such as age, or/as well as the relationship status/ partner can influence body image and therefore HRQOL and the risk of distress [30].

Obesity plays an important role in the link between negative body image and quality of life as metabolic alterations related to obesity are central to the pathogenesis of PCOS, with insulin resistance and obesity occurring more frequently in PCOS patients compared to age-matched populations [31]. These metabolic factors not only intensify PCOS symptoms but also impact the overall prognosis, as individuals with PCOS face a higher lifetime risk of conditions like gestational diabetes and type II diabetes mellitus (T2DM) [31]. Notably, some individuals with PCOS maintain a normal weight, with or without insulin resistance. The prevalence of obesity in women with PCOS has risen from 51% in the 1990s to 74% in later decades [4]. Even in the absence of obesity, individuals with PCOS are at risk for disorders related to body image, which may independently contribute to reduced quality of life. This highlights that PCOS is not limited to obese individuals, emphasizing the importance of addressing body image concerns in all PCOS patients and underscoring the complexity of metabolic factors in this disorder [8]. However, the exact impact of these metabolic disruptions on HRQOL in PCOS remains incompletely understood. The collective of our study had an increased mean BMI (31.3 kg/m^2^). Subsequent analysis showed that a BMI in the pathological range was associated with low quality of life, depression, and anxiety. In the multivariate analysis, this result remained statistically significant.

The primary approach for treating PCOS and obesity in PCOS patients is weight loss achieved through lifestyle interventions, which have been demonstrated to alleviate all symptoms associated with the syndrome. Research indicates that weight loss enhances HRQOL and diminishes symptoms of anxiety and depression in women with PCOS [4, 32]. To lose weight, a healthy diet and regular exercise are recommended. The superiority of one specific diet over another has not yet been proven [4]. However, other therapeutic approaches, such as cognitive behavioral therapy, can also reduce dissatisfaction with body image, anxiety, and depression in women with PCOS; therefore, the use of this therapy can be considered [33].

However, it must not be forgotten that the connection between body image and mental state is a reciprocal system, where each element influences the other. Individuals who are dissatisfied with their bodies may have lower self-esteem, avoid social situations, and feel restricted in their social lives. A negative perception of one’s body can be a risk factor for distress. These symptoms can range from feelings of hopelessness and worthlessness to severe depressive episodes.

On the other hand, distress can also negatively affect body image. Depressive symptoms can lead to increased dissatisfaction with one’s body and further diminish self-esteem. This can lead to a vicious cycle in which distress affects body image and vice versa [27, 34]. And the social element should also be considered: The pressure to meet beauty standards and the stigma of visible PCOS symptoms can exacerbate negative feelings and body image dissatisfaction plays a crucial role in the onset and perpetuation of diverse eating disorders [4, 35]. A recent systematic review and meta-analysis demonstrated a robust correlation between PCOS and a heightened prevalence of eating disorders [36].

The findings from our study underscored the necessity for a comprehensive approach to treating PCOS, which also addresses the psychological and emotional aspects of the condition. Unfortunately, screening women with PCOS for negative body image is not widespread practice. This could be due to the fact that screening might affect resources, including the duration of consultations [4]. Physicians and healthcare professionals should be aware of the potential impacts of body image disturbances on HRQOL and offer appropriate interventions to support affected patients. This may involve integrating psychological counseling, support groups, or physical exercise programs to promote a positive body image.

The interaction between obesity and psychological well-being in PCOS underscores the need for an integrated treatment approach that addresses not only metabolic and endocrine abnormalities but also mental health and lifestyle factors. Addressing obesity through lifestyle interventions, nutritional guidance, and mental health support can, therefore, play a crucial role in improving overall quality of life and psychological resilience in women with PCOS.

### Strengths and limitations

One limitation of this study was the absence of a control group, which restricted our ability to compare the outcomes and limited the generalizability of our findings. The method of enrollment utilized may have introduced bias into the study cohort. The survey was disseminated in hospitals and shared on social media platforms. Consequently, it is conceivable that younger and more educated PCOS patients may have been more inclined to participate due to their superior internet access and information availability. This circumstance could elucidate the low representation of postmenopausal patients within this group. Such a limitation in the study population had the potential to introduce biases into the findings. Hence, a critical evaluation of the sample’s representativeness regarding the broader population of PCOS patients is imperative for appropriately gauging the generalizability of the study’s conclusions.

Although each participant had to generate a personalized code, multiple participations by participants cannot be ruled out. This had the potential to impact the reliability and trustworthiness of the data. There was an ambiguity regarding whether the inaccurately marked or unanswered questions in this web-based survey reflected a genuine lack of knowledge or if certain responses were left unchecked due to time constraints or lack of diligence.

It was also uncertain whether all individuals in this survey truly suffer from PCOS. A part of the participants did not meet the ESHRE criteria but claimed to have received the diagnosis from a healthcare professional. Moreover, the assessment of androgenization is particularly susceptible to subjective bias, which cannot be ruled out.

On the other hand, this type of enrollment resulted in the inclusion of many PCOS patients in this study within a brief period. The power estimation for the number of participants was significantly exceeded, thus contributing to the robust statistical significance of this study. Additionally, a high number of potential confounders were assessed in this study, for example by assessing disorders of the metabolism of the participants. It is already known that individuals who are overweight are at an increased risk of experiencing depression [37]. These and many parameters were queried and included in the analysis, thus enhancing the robustness of the results of this study.

## Conclusion

Our findings reveal that obesity and a negative body image are interconnected factors that adversely affect both HRQOL and mental health in women with PCOS. Healthcare professionals should recognize the negative effects of obesity and poor body image in patients with PCOS and proactively provide effective treatment options.

## Data Availability

The data that support the findings of this study are not openly available due to reasons of sensitivity and are available from the corresponding author upon reasonable request. Data are located in controlled access data storage at Universitätsmedizin Mainz.
